# Early-Life Diet Diversity and the Subsequent Risk of Inflammatory Bowel Disease: Findings From Two Scandinavian Birth Cohorts

**DOI:** 10.1093/ibd/izae210

**Published:** 2024-09-14

**Authors:** Annie Guo, Johnny Ludvigsson, Elin M Hård af Segerstad, Anne Lise Brantsæter, Björn Andersson, Ketil Størdal, Karl Mårild

**Affiliations:** Department of Pediatrics, Institute of Clinical Sciences, Sahlgrenska Academy, University of Gothenburg, Gothenburg, Sweden; Crown Princess Victoria Children’s Hospital, Region Östergötland, Linköping, Sweden; Division of Pediatrics, Department of Biomedical and Clinical Sciences, Linköping University, Linköping, Sweden; Department of Pediatric Research, Oslo University Hospital, Oslo, Norway; Department of Food Safety and Centre for Sustainable Diets, Norwegian Institute of Public Health, Oslo, Norway; Bioinformatics and Data Centre, Sahlgrenska Academy, University of Gothenburg, Gothenburg, Sweden; Department of Pediatric Research, Oslo University Hospital, Oslo, Norway; Institute of Clinical Medicine, University of Oslo, Oslo, Norway; Department of Pediatrics, Institute of Clinical Sciences, Sahlgrenska Academy, University of Gothenburg, Gothenburg, Sweden; Department of Pediatrics, Queen Silvia Children’s Hospital, Gothenburg, Sweden

**Keywords:** childhood diet, Crohn’s disease, ulcerative colitis, ABIS, MoBa

## Abstract

**Background:**

Diet diversity in early childhood promotes microbial diversity, influences the developing immune system, and has been linked to a reduced risk of immune-mediated diseases. This study aimed to determine the association between childhood diet diversity and later inflammatory bowel disease (IBD), for which data are limited.

**Methods:**

Questionnaire data from the population-based birth cohorts All Babies in Southeast Sweden (ABIS) and the Norwegian Mother, Father, and Child Cohort (MoBa), including participants from Southeast Sweden and Norway, were used to estimate a diet diversity score at ages 1 and 3 years. This score represents the diversity of intakes across 5 food groups comprising 11 subgroups. A higher score signifies higher diet diversity. We used linked health registry data to identify IBD diagnoses up to the year 2021. Cox regression and random-effect models were used to estimate pooled hazard ratios (aHRs) adjusted for sociodemographics, breastfeeding, and early-life antibiotic use.

**Results:**

Among 81 272 children with 1 304 325 person-years of follow-up, 307 developed IBD. Diet diversity at ages 1 and 3 years was in pooled analyses not associated with later IBD (per one-unit increase, aHR = 0.96 [95% CI = 0.81-1.14] and aHR = 0.96 [95% CI = 0.83-1.11]). In MoBa, but not ABIS, a higher diet diversity at 1 and 3 years of age was inversely associated with ulcerative colitis (UC) (per one-unit increase, aHR = 0.78 [95% CI = 0.66-0.94] and aHR = 0.78 [95% CI = 0.65-0.95]). Still, pooled aHRs for UC as well as Crohn’s disease approximated one.

**Conclusions:**

In this prospective study of 2 Scandinavian birth cohorts, no association was observed between early-life diet diversity and the subsequent risk of IBD.

**Key Messages**

**What is already known?**

Exposure to diet in early life and adulthood has been hypothesized to influence the risk of inflammatory bowel disease (IBD), but the role of diet diversity in early childhood is unclear.

**What is new here?**

In this prospective cohort study, having a high diet diversity at 1 and 3 years of age was not associated with the later risk of IBD or its subtypes.

**How can this help patient care?**

This study brings attention to the potential role of early-life diet for IBD development and, along with prior literature, suggests that diet quality rather than diet diversity in early life influences the risk of IBD.

## Introduction

The diet diversity implies consuming a variety of different foods. To support overall health, it is advised that children aged 6 months and older incorporate a diverse assortment of animal-source foods, fruits, vegetables, dairy, and grains in their diet.^1,2^ A poor diet diversity contributes to nutrient deficiencies and chronic diseases,^2-4^ while a greater variety in the first year of life has been associated with heightened microbial diversity in infants.^5^

Inflammatory bowel disease (IBD) is an immune-mediated disease comprising ulcerative colitis (UC), Crohn’s disease (CD), and IBD-unclassified (IBD-U). After a notable increase in recent decades, it now affects some 0.5% of Western populations, signifying its emergence as a global disease.^6^ This rise in IBD incidence parallels our changing lifestyle and emphasizes the key role of the environment in disease development.^7^ Intestinal dysbiosis and reduced microbial diversity likely also play a role in IBD development.^8^ Because the infant gut microbiome does not stabilize until 3 years of age,^9,10^ particularly early-life diets (eg, healthy dietary patterns^11,12^ and specific foods)^12,13^ have been suggested to modify the risk of IBD.^12,14^

The favorable influence of diet diversity on the microbiome composition^15^ suggests that a diverse diet in early life may also shape the risk of later IBD. However, there are few studies on the relationship between early-life diet diversity and the risk of later disease development overall, with no previous data specifically on IBD risk (Supplementary Table S1). Therefore, the aim of this study was to examine the association between early-life diet diversity and the risk of IBD using data from 2 Scandinavian birth cohorts.

## Materials and Methods

### Study Population

**Figure 1.**
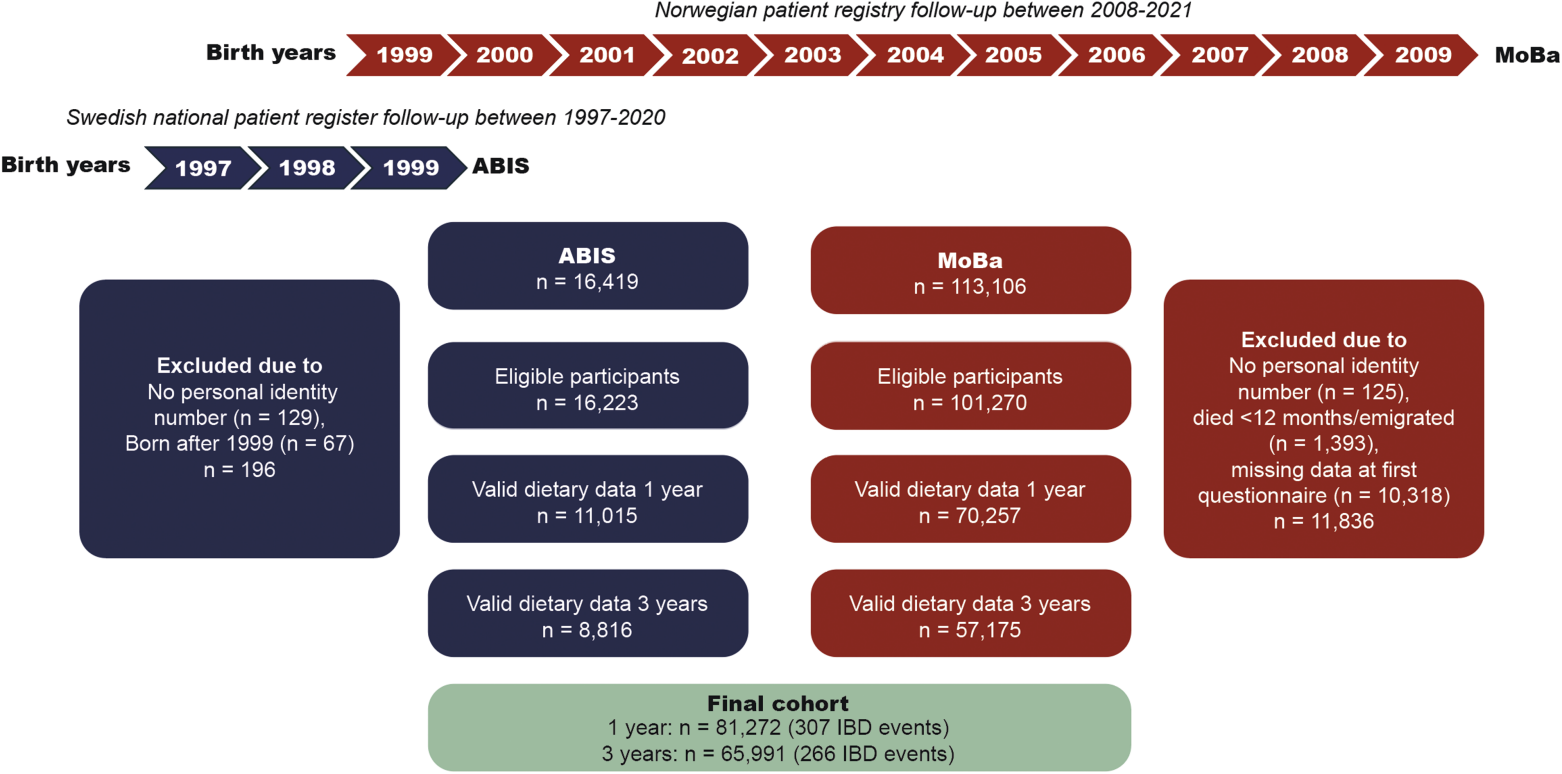
Flowchart of study participants from the All Babies in Southeast Sweden (ABIS) and the Norwegian Mother, Father, and Child Cohort Study (MoBa) with data from enrollment questionnaires (administered at birth and gestational week 15, respectively) and parent-reported data on at least one food item at age 1 year. Data on stillbirths and infant deaths were not available in ABIS. Valid dietary data were defined as having data on at least one food or beverage item.

We included prospective data from two population-based cohort studies: All Babies in Southeast Sweden (ABIS) and the Norwegian Mother, Father, and Child Cohort (MoBa). ABIS invited about 21 700 families living in Southeast Sweden when their child was born between October 1997 and October 1999. More than 16 000 children were included (participation rate 79%).^16^ MoBa enrolled more than 95 000 pregnant women across all of Norway between 1999 and 2008 and 41% of those invited consented to participate. Children participating in both cohorts were followed from birth throughout adolescence and early adulthood by repeated questionnaires and through linkage to national health registries (Supplementary Tables S2 and S3).^17–20^ This study was restricted to participants with valid personal identity numbers (needed for linkage to registries) who reported child food frequency data at the child’s age 1 and 3 years (Figure 1). In line with previous studies,^12,21^ we included children with reported data on at least one food item, ie, reported intake frequency on at least one food-related question (about 98% of all children in ABIS and MoBa had available data on >50% of the food items and <2% had data on 1 or 2 items only).

### Diet Diversity in Early Life

Data were collected from comprehensive questionnaires that, in addition to questions about child health and development, contained 40-50 items about the child’s current food and beverage intake at both 1 and 3 years of age (12 and 30-36 months [ABIS] and 18 and 36 months [MoBa]). The questionnaires included information on the child’s intake of meat, fish, vegetables, fruits, bread, porridge, and beverages. The parents reported the child’s intake at predefined frequencies ranging from never to 6-10 times daily. All responses were converted to reflect weekly intakes.

Based on previous studies examining diet diversity related to different adverse health outcomes^22,23^ and recommendations provided by the World Health Organization^2^ and the US Department of Agriculture,^1^ we developed a diet diversity score (DDS). The DDS captures variation in food intake across 11 food items. Frequencies of ≥2 intakes weekly were considered in the score (Supplementary Material page 2 and Supplementary Table S3).

To measure the diet diversity of recommended food groups,^1,2^ the 11 food items were weighted into five major food groups of “Grains, roots, tubers,” “Vegetables,” “Fruits,” “Dairy,” and “Animal Proteins” separately at both 1 and 3 years of age (Supplementary Table S4). Each major food group had a maximum score of 2 points. To be allocated a maximum score, the reported intake of each contributing food item needed to be at least 7 intakes/week. Less frequent intakes resulted in a proportionally lower score. For example, a child consuming at least 7 intakes/week of each food item bread, home-made porridge, baby cereals, and potatoes (included in the major food group “Grains, roots and tubers”) at 1 year would receive a maximum score of 2 in “Grains, roots and tubers.” Instead, only intake of bread with the same frequency, but neither home-made porridge, baby cereals, or potatoes would result in a score of 0.5 for “Grains, roots, and tubers.” The food groups “Vegetables” and “Fruits” each only included one food item and children with ≥7 intakes/week received a maximum score of 2 points while those with a less frequent intake received a continuous score between 0 and 1.5. Finally, the 5 major food groups were summarized into a total DDS ranging from 0 to 10 points, where a higher score reflects a higher diet diversity across the food groups. To consider the different questionnaires used in ABIS and MoBa, the DDS was divided into levels of diet diversity based on tertiles in each cohort separately reflecting low (reference), medium, and high diet diversity. To analyze the risk per unit increase in DDS, the DDS was modeled as a continuous score. To align with dietary guidelines and previous publications,^2^ the DDS did not consider high-sugar foods and beverages (eg, sugar-sweetened beverages and confectionary).

### Inflammatory Bowel Disease

The primary outcome of this study was any IBD, including the subtypes of CD, UC, and IBD-U. As secondary outcomes, the subtypes CD and UC were examined separately. However, a priori, we did not include IBD-U as a separate outcome because of the limited number of cases and higher risk of misclassification. We defined IBD as ≥2 International Classification of Diseases, Tenth Revision (ICD-10) entries in the Swedish and Norwegian national patient registries captured by December 31, 2020 (ABIS) and December 31, 2021 (MoBa) (Supplementary Table S3). This outcome definition has a positive predictive value of 93%-94% in Sweden and Norway.^16,24^ We used subtype specific ICD-10 codes to define CD and UC. The national patient registries contain nationwide data from inpatient and hospitalized-based outpatient care in Sweden and Norway.^17,18^

### Other Data

To account for factors that may influence the association between diet diversity and IBD risk,^12,25,26^ we preselected the following covariate data from questionnaires or health registries: Parental IBD, parental origin (defined as the mother’s native language (MoBa) or the parent’s country of birth [ABIS]), maternal education level, age, immune-mediated comorbidities (based on previous literature,^25^ this included type 1 diabetes, autoimmune thyroid disease, or rheumatoid arthritis), smoking in pregnancy, and delivery mode. Child factors included sex, birth weight, gestational age, full breastfeeding duration^2^ and antibiotic use by 1 year of age. We also included covariate data on the child’s diet quality at age 1 year, previously defined using a healthy eating index (HEI) score.^12^ All covariates were categorized as shown in Table 1 and Supplementary Table S3.

**Table 1.** Characteristics of the participants in All Babies in Southeast Sweden (ABIS) and Norwegian Mother, Father, and Child Cohort (MoBa) presented by level of diet diversity at 1 year of age.

	Diet diversity by 1 year of age					
	ABIS (*n* = 11 105)			MoBa (*n* = 70 257)		
	Low (*n* = 3631)	Medium (*n* = 3682)	High (*n* = 3702)	Low (*n* = 23 139)	Medium (*n* = 24 056)	High (*n* = 23 062)
*Diet diversity score by 1 age year*						
Mean (SD)	4.8 (1.2)	6.8 (0.3)	7.8 (0.4)	4.8 (0.9)	6.4 (0.3)	7.7 (0.5)
*Diet diversity score by age 3 years*						
Mean (SD)	5.6 (1.5)	6.2 (1.3)	6.6 (1.2)	5.7 (1.3)	6.5 (1.1)	7.1 (1.0)
*Child’s sex*						
Female	1797 (49.5)	1753 (47.6)	1740 (47.0)	11 187 (48.3)	11 843 (49.2)	11 304 (49.0)
Male	1834 (50.5)	1929 (52.4)	1962 (53.0)	11 952 (51.7)	12 213 (50.8)	11 758 (51.0)
*Parental origin* ^a^						
Sweden/Norway	3099 (85.3)	3273 (88.9)	3371 (91.1)	20 486 (88.5)	21 218 (88.2)	19 967 (86.6)
*Missing*	105 (2.9)	70 (1.9)	65 (1.8)	601 (2.6)	90 (2.5)	591 (2.6)
*Maternal education level* ^b^						
≤11 years	408 (11.2)	208 (5.6)	173 (4.7)	2168 (9.4)	1233 (5.1)	830 (3.6)
12 years	2143 (59.0)	1964 (53.3)	1865 (50.4)	7739 (33.4)	6332 (26.3)	5119 (22.2)
≥13 years	969 (26.7)	1442 (39.2)	1596 (43.1)	12 907 (55.8)	16 224 (67.4)	16 847 (73.1)
*Missing*	111 (3.1)	68 (1.8)	68 (1.8)	325 (1.4)	267 (1.1)	266 (1.2)
*Parental IBD* ^c^						
Yes	51 (1.4)	34 (0.9)	56 (1.5)	573 (2.5)	580 (2.4)	556 (2.4)
*Maternal comorbidities* ^d^						
Yes	114 (3.1)	132 (3.6)	139 (3.8)	906 (3.9)	976 (4.1)	971 (4.2)
*Full breastfeeding duration*						
<4 months	909 (25.0)	848 (23.0)	919 (24.8)	9698 (41.9)	9398 (39.1)	8541 (37.0)
4-6 months	979 (27.0)	1172 (31.8)	1251 (33.8)	9799 (42.4)	10 510 (43.7)	10 248 (44.4)
>6 months	566 (15.6)	511 (13.9)	441 (11.9)	2693 (11.6)	3294 (13.7)	3457 (15.0)
*Missing*	1177 (32.4)	1151 (31.3)	1091 (29.5)	949 (4.1)	854 (3.6)	816 (3.5)
*Maternal smoking in pregnancy*						
Yes	465 (12.8)	270 (7.3)	274 (7.4)	2366 (10.2)	1746 (7.3)	1404 (6.1)
*Missing*	111 (3.1)	68 (1.8)	68 (1.8)	298 (1.3)	284 (1.2)	284 (1.2)
*Mode of delivery*						
Vaginal	2952 (81.3)	2981 (81.0)	2974 (80.3)	19 798 (85.6)	20 554 (85.4)	19 630 (85.1)
*Missing*	278 (7.7)	286 (7.8)	304 (8.2)	0 (0.0)	0 (0.0)	0 (0.0)
*Maternal age at delivery* ^e^						
<25	702 (19.3)	449 (12.2)	404 (10.9)	3015 (13.0)	2074 (8.6)	1548 (6.8)
25-34	2454 (67.6)	2718 (73.8)	2736 (73.9)	16 385 (70.8)	17 674 (73.4)	16 997 (73.7)
35-44	406 (11.2)	445 (12.1)	508 (13.7)	3730 (16.1)	13 571 (17.9)	4501 (19.6)
*Missing*	69 (1.9)	70 (1.9)	54 (1.5)	9 (0.0)	10 (0.0)	16 (0.1)
*Birth weight (grams)* ^f^						
Mean (SD)	3583 (551)	3579 (536)	3578 (543)	3573 (583)	3580 (576)	3560 (576)
*Missing*	51 (1.4)	34 (0.9)	25 (0.7)	7 (0.0)	15 (0.0)	17 (0.0)
*Gestational age (weeks)* ^g^						
Mean (SD)	39.7 (1.7)	39.7 (1.8)	39.8 (1.6)	39.4 (1.9)	39.4 (1.9)	39.4 (1.9)
*Missing*	89 (2.5)	57 (1.5)	48 (1.3)	92 (0.4)	105 (0.4)	92 (0.4)
*Child’s diet quality by 12 months* ^h^						
Low	2235 (61.6)	1442 (39.2)	874 (23.6)	11 608 (50.2)	7093 (29.5)	2821 (12.2)
Medium	1034 (28.5)	1167 (31.7)	1236 (33.4)	8189 (35.4)	9812 (40.8)	8763 (38.0)
High	362 (10.0)	1073 (29.1)	1592 (43.0)	3342 (14.4)	7151 (29.7)	11 478 (49.8)
*Child’s antibiotic use 0-12 months* ^i^						
Yes	1266 (34.9)	1255 (34.1)	1263 (34.1)	2618 (11.3)	2683 (11.2)	2567 (11.1)
*Missing*	185 (5.1)	93 (2.5)	85 (2.3)	60 (0.3)	45 (0.2)	33 (0.1)

^a^Parent’s native language (MoBa) / parent’s country of birth (ABIS).

^b^Captured in mid-pregnancy.

^c^Defined as having at least one parent with IBD.

^d^Type 1 diabetes (insulin-treated diabetes before or during pregnancy [MoBa] or type 1 diabetes/insulin-treated diabetes [ABIS]), autoimmune thyroid disease, or rheumatoid arthritis.

^e^<15 years were defined as missing in ABIS (not applicable in MoBa), and >44 years were set as missing in both cohorts.

^f^<270 or >6999 g were set as missing. ^j^<22 or >45 weeks were changed to missing.

^g^<22 or >45 weeks were changed to missing.

^h^Calculated by using a modified Healthy Eating Index score described by Guo et al.^12^.

^i^Categorized as exposed or unexposed.

ABIS, all babies in Southeast Sweden; CD, Crohn’s disease; IBD, inflammatory bowel disease; IQR, interquartile range; MoBa, The Norwegian mother, father, and child cohort study; SD, standard deviation; UC, ulcerative colitis.

### Statistical Analysis

The Cox proportional hazard regression model was used to estimate cohort-specific adjusted hazard ratios (aHRs) accounting for the child’s sex, parental IBD and origin, maternal education level at delivery, and maternal comorbidities. We separately analyzed diet diversity when the child was 1 and 3 years old with DDS modeled as a continuous and trichotomous (tertile) exposure variable. The follow-up time was set from the exposure (at 1 or 3 years of age) to the first IBD diagnosis or censoring at the end of data capture, whichever occurred first. The proportional hazard assumption was found valid for all IBD analyses at 1 and 3 years of age (except for DDS as a continuous exposure variable in MoBa at 3 years of age) after assessments using Schoenfeld residuals and time-interaction analyses. The cohort-specific estimates were pooled using a random-effects model.^27^ Incidence rates for IBD, CD, and UC were calculated using Poisson regression. All analyses were performed in R Statistical Software (versions 4.1.3 and 4.2.2) using survival, survminer, meta, and metaphor packages. We performed complete case analyses, wherein individuals with missing information on covariate data were excluded.

### Sensitivity Analyses

To test the robustness of our main analyses, we performed several sensitivity analyses in which we additionally adjusted for (1) full breastfeeding duration, (2) maternal smoking in pregnancy, (3) perinatal factors (delivery mode, maternal age, birth weight, gestational age), (4) the child’s diet quality at age 1 year, (5) the child’s antibiotic use by 1 year, and (6) the intake of sugar-sweetened beverages at 12 months of age (Supplementary Table S3). Using the Spearman rank correlation coefficient, we estimated the correlation between DDS at 1 and 3 years of age as well as the correlation between DDS and HEI at each age separately. We also assessed the change in DDS from 1 to 3 years of age. To examine the risk of diet diversity and childhood-onset IBD, we restricted our analyses to a follow-up time of <18 years.

## Results

In this study of 2 Scandinavian birth cohorts, 81 272 participants were followed for 234 071 and 1 070 254 person-years in ABIS and MoBa, respectively, corresponding to a total follow-up time of 1 304 325 person-years (Supplementary Table S5). The median follow-up time from the child’s age of 1 year was 21.3 years (ABIS) and 15.2 years (MoBa). In this study, 307 children developed IBD (131 CD, 97 UC, 79 IBD-U).

Compared to children with the lowest diet diversity at age 1 year, those with higher levels had better diet quality, and their mothers had a higher education level and were older at delivery (Table 1; Supplementary Table S6 presents characteristics divided by children with and without IBD). Approximately half of the children remained at the same level of DDS from age 1 to 3 years (Supplementary Table S7). Of participants who changed their level of diet diversity from age 1 to 3 years of age, approximately half had a poorer diet diversity, and the other half had an increased diversity level at the age of 3 years. The correlation between DDS at 1 and 3 years of age was *r* = 0.27 in ABIS and *r* = 0.40 in MoBa. The correlation between DDS and HEI at 1 year of age was *r* = 0.35 in ABIS, and *r* = 0.38 in MoBa, while the correlations at 3 years of age were identical in both cohorts (*r* = 0.34).

### Child Diet Diversity and the Subsequent IBD Risk

**Figure 2.**
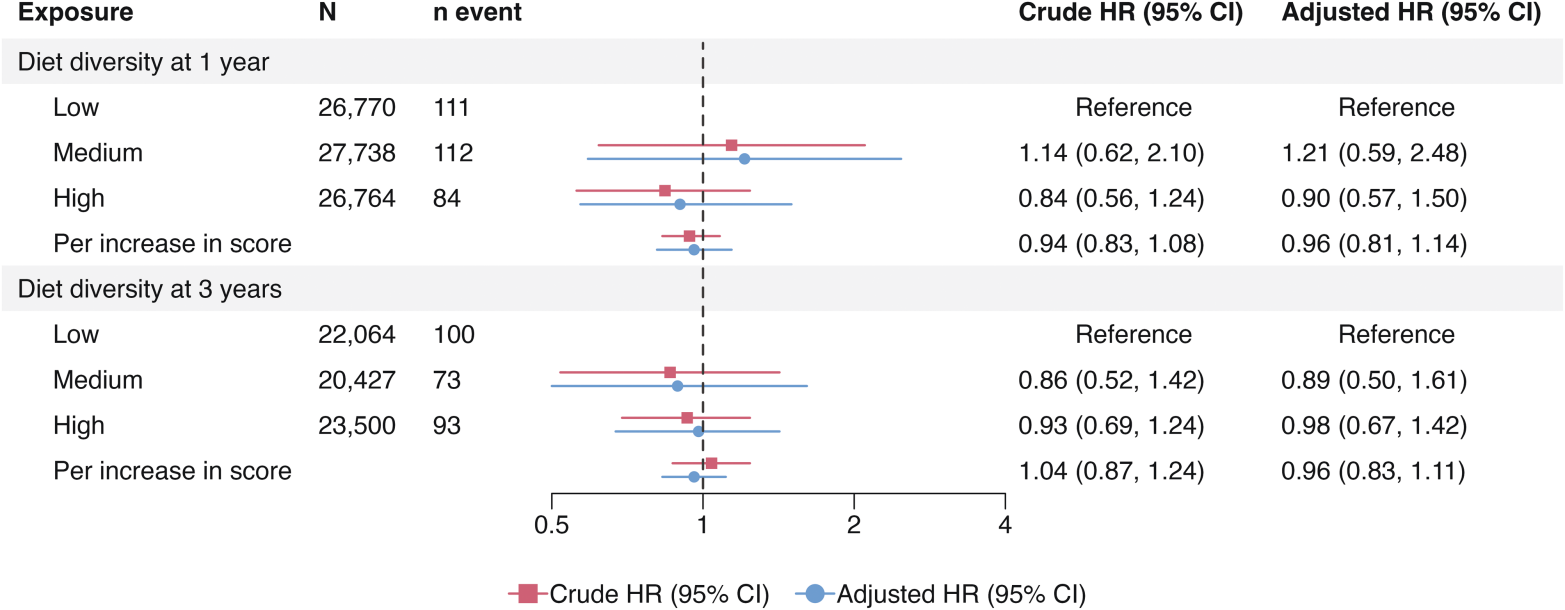
Pooled hazard ratios for inflammatory bowel disease (IBD) according to diet diversity at 1 and 3 years of age adjusted for the child’s sex, parental IBD, origin, maternal education level at delivery, and comorbidities. Per increase in score refers to per one unit increase. CI, confidence interval; HR, hazard ratio.

**Figure 3.**
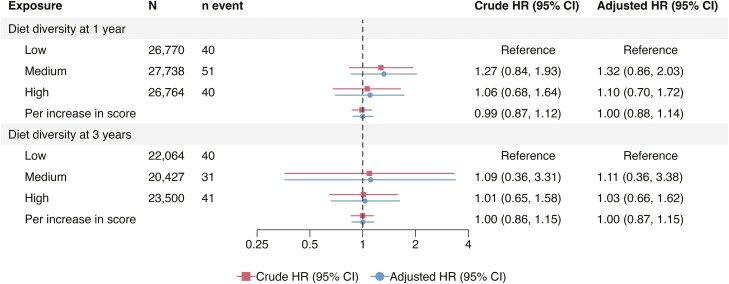
Pooled hazard ratios for Crohn’s disease according to diet diversity at 1 and 3 years of age adjusted for the child’s sex, parental inflammatory bowel disease, origin, maternal education level at delivery, and comorbidities. Per increase in score refers to per one unit increase. CI, confidence interval; HR, hazard ratio.

**Figure 4.**
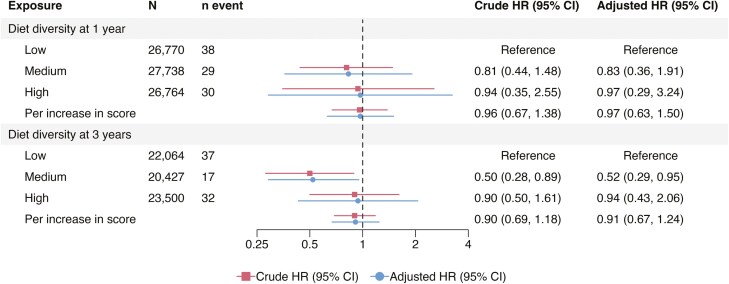
Pooled hazard ratios for ulcerative colitis according to diet diversity at 1 and 3 years of age adjusted for child’s sex, parental inflammatory bowel disease, origin, maternal education level at delivery, and comorbidities. Per increase in score refers to per one unit increase. CI, confidence interval; HR, hazard ratio.

The pooled analyses indicated that there was no association between the diet diversity at age 1 year and later development of IBD (high vs low diet diversity, aHR = 0.90 [95% CI = 0.57-1.50] and per unit increase in DDS, aHR = 0.96 [95% CI = 0.81-1.14]; Figure 2) when adjusting for the child’s sex, parental IBD and origin, as well as the maternal education level and comorbidities. Additionally, the pooled aHRs for diet diversity at age 1 year and CD and UC risk approximated 1 (Figures 2-4).

In cohort-specific analyses, high versus low diet diversity at age 1 year yielded aHRs for IBD of 1.26 (95% CI = 0.68-2.33) in ABIS and 0.73 (95% CI = 0.52-1.02) in MoBa (Supplementary Table S8). When treating DDS as a continuous exposure variable (per unit increase), a higher diversity score at 1 year was associated with a lower risk of later IBD and UC in MoBa (aHR = 0.89 [95% CI = 0.81-0.98] and aHR = 0.78 [95% CI = 0.66-0.94, respectively]), but not in ABIS (IBD, aHR = 1.06 [95% CI = 0.90-1.25]; UC = aHR 1.23 [95% CI = 0.94-1.60]; Supplementary Tables S8 and S9). The cohort-specific analyses revealed no association for CD (Supplementary Table S10).

For diet diversity at 3 years, the pooled analyses showed no association with either IBD (high vs low, aHR = 0.98 [95% CI = 0.67-1.42], and per unit increase in DDS, aHR = 0.96 [95% CI = 0.83-1.11]; Figure 2) or CD (high vs low, aHR = 1.03 [95% CI = 0.66-1.62] and per unit increase in DDS, aHR = 1.00 [95% CI = 0.87-1.15]; Figure 3). Comparable null results were observed for the UC risk, except for an inverse association when comparing medium versus low diet diversity (pooled aHR = 0.52 [95% CI = 0.29-0.95]; Figure 4). The cohort-specific analyses of high versus low diversity at 3 years yielded an aHR for IBD of 1.31 (95% CI = 0.70-2.45) in ABIS, and 0.86 (95% CI = 0.62-1.20) in MoBa (Supplementary Table S8). Similar to the 1-year analyses, a high versus low diversity score at 3 years of age was associated with a reduced risk of later UC in MoBa (per unit increase, aHR = 0.78 [95% CI = 0.65-0.95]), but not in ABIS (aHR = 1.08 [95% CI = 0.84-1.39]; Supplementary Table S9).

### Sensitivity Analyses

The analysis presented in Supplementary Table S11 shows that there were no associations between diet diversity and the risk of IBD after additionally adjusting for either the full breastfeeding duration, maternal smoking during pregnancy, perinatal factors, the child’s diet quality, antibiotic use, or intake of sugar-sweetened beverages by 1 year of age. Our analyses, restricted to childhood-onset IBD (ie, <18 years), supported the results from our main analyses (Supplementary Table S12).

## Discussion

This study conducted pooled analyses of over 80 000 children from 2 Scandinavian birth cohorts. The results revealed no associations between diet diversity at either 1 or 3 years of age and the subsequent risk of IBD, CD, or UC. These null findings were confirmed in sensitivity analyses further adjusted for the potential confounding effects of full breastfeeding duration, early-life diet quality, and antibiotic use. While a higher DDS was inversely associated with IBD and UC risk in MoBa but positively associated with IBD in ABIS, the lack of consistency across cohorts argues against a causal relationship. Instead, this study supports previous findings^12^ that a healthy early-life diet rather than an overall diverse diet may modulate the risk of later development of IBD.

Diet has been suggested to be an important determinant of the gut microbiome’s function, abundance, and diversity.^28^ The gut microbiome can in early life modulate the immune system and cause gut dysbiosis, a reduction of commensals, and impaired diversity, which has been described as a risk factor for IBD.^28^ Because the gut microbiome undergoes significant changes until stabilizing around 3 years of age,^9^ early-life diets may be particularly important for the subsequent IBD risk. Although the importance of diet diversity in achieving adequate nutrient intake is highlighted,^1-3^ studies on its association with subsequent childhood disease development are limited. While some studies have suggested a protective association between diet diversity at 6 months of age and allergic rhinitis,^22^ others have not observed any association between diet diversity and childhood eczema.^29^

One likely cause of this knowledge gap is the limited availability of large, unselected cohorts needed to provide reliable and sufficiently powered risk estimates. Also, such examinations require prospectively collected comprehensive dietary data and long-term follow-up to identify events of IBD. Furthermore, apart from WHO’s minimum dietary diversity index,^30^ there is a lack of a firmly established definition of early-life diet diversity to be used in the investigation of childhood-onset diseases. While some studies have defined diet diversity to measure the introduction of complementary foods in infancy,^31,32^ others have calculated a diet diversity index^23,29^ or included diet diversity as a component of a healthy diet after weaning.^33^

Our previous study observed an inverse association between a high diet quality at 1 year of age and the subsequent IBD risk.^12^ Together with the present study, the data suggest that a healthy diet in early life, characterized by higher intakes of fruits, vegetables, fish, and dairy, and reduced consumption of sugary foods and drinks and meat, rather than a diverse food intake, is of importance for IBD risk. However, the level of diet diversity was not consistent throughout childhood; about half of the participants either increased or decreased their diet diversity from 1 to 3 years of age. This is consistent with data on the stability of overall dietary intake throughout childhood, showing changes over time, however, with common traits in dietary quality.^34,35^ We, therefore, cannot rule out the possibility that diet diversity later in childhood (eg, in young adulthood closer to the onset of IBD) may have a stronger influence on the IBD risk.

As in any study reporting null findings, we acknowledge the risk of a type 2 error. Although early-life diet was in this study prospectively recorded from comprehensive and repeated questionnaires, data on portion sizes and additional food items that allow for a more precise estimation of diet diversity would have reduced the risk of exposure misclassification, which might otherwise bias estimates towards the null.^36^ This study was unable to account for the level of food processing or consumption of processed foods and food additives. We acknowledge that such information would be an important aspect to consider in future studies investigating diet and the risk of IBD. Also, our sample size does not allow us to rule out more modest associations with IBD, particularly for UC and CD subtypes.

This study of >80 000 children represents the first attempt to evaluate the relationship between early-life dietary variety and the subsequent risk of developing IBD. Using 2 prospective population-based birth cohorts enabled us to test the consistency of associations across the 2 cohorts and include over 300 IBD events. Our outcome algorithm of IBD has previously been compared to medical records and shown a positive prediction value of ≥93% for the diagnosis of IBD in Sweden and Norway.^16,24^ We had comprehensive information on various health and lifestyle factors that could be potential confounders that allowed for performing rigorous sensitivity analyses adjusted for parental socioeconomic status, breastfeeding duration, early-life diet, and antibiotic use. Although the food-specific questions were not intended to specifically capture diet diversity, these questions have been broadly used to assess the association between early-life diet and childhood health outcomes.^12,21,33^

The generalizability of our findings to countries of different food habits or regions with a lower prevalence of IBD remains uncertain, given that this study was conducted in a Western population/high-income country with a high prevalence of the disease. Despite obtaining consistent findings across all sensitivity analyses, the possibility of unmeasured variables impacting our results cannot be disregarded. In addition, the limited number of CD and UC cases may have increased the risk of type 2 errors. We acknowledge that the follow-up time of the ABIS and MoBa cohorts limits the number of identified IBD cases (the median age at the end of follow-up was 21.3 and 15.2 years in ABIS and MoBa) and that our analyses are predominantly restricted to pediatric IBD patients. Also, this study does not include biological data, and we primarily measured variation across rather than within major food groups.

In this study of 2 Scandinavian birth cohorts, we observed that diet diversity at neither 1 nor 3 years of age was associated with IBD risk. Other large-scale prospective studies with even longer follow-ups and more detailed data on dietary factors such as food processing and additives are needed to confirm these null findings.

## Supplementary Data

Supplementary data is available at *Inflammatory Bowel Diseases* online.

izae210_suppl_Supplementary_material

## Data Availability

All relevant data are included in the manuscript and its Supplementary Material. The lead authors (the manuscript’s guarantors) affirm that the manuscript is an honest, accurate, and transparent account of the study being reported; that no important aspects of the study have been omitted; and that any discrepancies from the study as planned (and, if relevant, registered) have been explained. The consent given by the participants does not allow the storage of data on an individual level in repositories or journals. Researchers who want access to data sets for replication should apply through helsedata.no. Access to data sets requires approval from the Regional Committee for Medical and Health Research Ethics in Norway and an agreement with MoBa.
