# Supplementary material for: Early-Life Diet Diversity and the Subsequent Risk of Inflammatory Bowel Disease: Findings From Two Scandinavian Birth Cohorts
Source: Inflamm Bowel Dis. 2024 Sep 14;31(6):1493–501. doi: 10.1093/ibd/izae210 (PMC12166299; doi:10.1093/ibd/izae210)
Supplement: izae210_suppl_Supplementary_material [file izae210_suppl_supplementary_material.docx]

**SUPPLEMENTARY TO**

*Guo et al. Early-life diet diversity and the subsequent risk of inflammatory bowel disease: Findings from two Scandinavian birth cohorts*

TABLE OF CONTENT

[SUPPLEMENTARY MATERIALS 2](#_Toc165980926)

[**Diet diversity score** 2](#_Toc165980927)

[SUPPLEMENTARY TABLES 3](#_Toc165980928)

[**Supplementary Table 1.** Previous literature investigating early-life diet diversity and childhood outcomes 3](#_Toc165980929)

[**Supplementary Table 2.** Description of All Babies in Southeast Sweden and the Norwegian Mother, Father and Child Cohort 4](#_Toc165980930)

[**Supplementary Table 3.** Data sources of variables used in ABIS and MoBa 5](#_Toc165980931)

[**Supplementary Table 4.** Description of the food categorization in the diet diversity score 6](#_Toc165980932)

[**Supplementary Table 5.** Follow-up and incidence of IBD, CD and UC in the ABIS and MoBa cohorts 7](#_Toc165980933)

[**Supplementary Table 6.** Study characteristics of participants in ABIS and MoBa presented by non-events and IBD cases by 1 year of age 8](#_Toc165980934)

[**Supplementary Table 7.** Tracking of diet diversity from 1 to 3 years of age in children with data at both time points^a^ 10](#_Toc165980935)

[**Supplementary Table 8.** Diet diversity at 1 and 3 years of age and risk of inflammatory bowel disease in ABIS and MoBa 11](#_Toc165980936)

[**Supplementary Table 9.** Diet diversity at 1 and 3 years of age and risk of Crohn’s disease in ABIS and MoBa 12](#_Toc165980937)

[**Supplementary Table 10.** Diet diversity at 1 and 3 years of age and risk of ulcerative colitis in ABIS and MoBa 12](#_Toc165980938)

**SUPPLEMENTARY MATERIALS**

## **Diet diversity score**

The Diet Diversity Score (DDS) was based on variation in the child’s weekly intakes of 11 food items within 5 food groups captured at 1 and 3 years of age (Supplementary Table 4). The DDS was a stepwise process calculated as follows:

1. Weekly intakes of food items were categorized into five food groups at age 1 year (*bread, home-made porridge, baby cereals, potatoes, vegetables, fruits, milk, yoghurt, cheese, meat, fish*) and 3 years (*bread, pasta, potatoes, vegetables, fruits, milk, yoghurt, cheese, meat, fish, egg*) separately.
2. Each food item was given a score from 0 to 1.

*<1.9 intakes/week = 0 points
2.0-2.9 intakes/week = 0.25 points
2.0-4.9 intakes/week = 0.50 points
5.0-6.9 intakes/week = 0.75 points
>7 intakes/week = 1 point*

1. Scores were summed up separately in each food group. As each food group constituted a different number of questions in the study questionnaires, the ll food groups were equally weighed in the DDS.

   The total score for each food group: (0-1 points) * 2 / number of included food items in the food group. The maximum score for each food group was 2.

   For the food groups *Vegetables* and *Fruits* that included one food item each, children with >7 intakes/week received a maximum score of 2 points while those with a less frequent intake received a continuous score between 0 and 1.5:

*Points in major food groups*

*<1.9 intakes = 0 points 0*

*2.0-2.9 intakes/week = 0.25 points 0.5*

*2.0-4.9 intakes/week = 0.50 points 1*

*5.0-6.9 intakes/week = 0.75 points 1.5*

*>7 intakes/week = 1 point 2*

1. The five food groups were summed up in the total DDS, and yielded a score from 0-10 points, where a higher score reflects a higher diet diversity across the food groups. DDS was also categorized in tertiles in ABIS and MoBa separately, indicating low, medium, and high diet diversity.

**SUPPLEMENTARY TABLES**

## **Supplementary Table 1.** Previous literature investigating early-life diet diversity and childhood-onset outcomes^a^

| Author, year (country) | Study design | Study population | Dietary data | Exposure | Outcome | Main Findings |
| --- | --- | --- | --- | --- | --- | --- |
| Nwaru, 2014 (Finland)^1^ | Cohort study | 3,142 children followed from 3-12 months of age | Dietary questionnaire | Food diversity | Allergy Asthma | Food diversity at 3 and 4 months of age was not associated with childhood wheezing, atopic eczema, or allergic rhinitis. At 6 months of age, a lower diversity of foods was associated with an increased risk of allergic rhinitis (>8 food items vs 0-4 food items, aOR 2.16 [95% CI 1.20-3.89]).  At 12 months of age, a lower diversity of foods was associated with an increased risk of asthma, wheezing, and allergic rhinitis. |
| Dello Russo, 2023 (8 European countries)^2^ | Cohort study | 3,035 children aged 6-11 years. | 24h dietary recall | DDS FVS | BMI Blood pressure Blood samples Waist circumference | No association was found between DDS and childhood obesity. The dietary diversity score, which consists of food groups, was a more accurate estimate than the food variety score, which only considered individual food items. |
|  |  |  |  |  |  |  |
| Venter, 2020 (United Kingdom)^3^ | Cohort study | 969 children followed from 3-12 months of age | Questionnaires | WHO DDS | Eczema Food allergy | No association was observed between diet diversity and eczema or food allergy. |

^a^ Did not consider studies assessing diet diversity in a developing country or focused on complementary food introduction.

CD, Crohn’s disease; DDS, dietary diversity score; FVS, food variety score; IBD, inflammatory bowel disease, UC, ulcerative colitis.

## **Supplementary Table 2.** Description of All Babies in Southeast Sweden and the Norwegian Mother, Father, and Child Cohort

|  | All Babies in Southeast Sweden (ABIS)^a^ | The Norwegian Mother, Father and Child Cohort (MoBa)^b^ |
| --- | --- | --- |
| Place of recruitment | Southeast Sweden | Norway |
| Included participants | 16,419 | 113,106 |
| Participation rate | 79% | 41% |
| Year of birth | 1997-1999 | 1999-2009 |
| Time of administered parent-reported questionnaires | At the child’s birth, and 12 and 30-36 months after birth | Gestational week 15, 22, 30, and 6, 18 and 36 months after birth |
| Linked health registers | Swedish National Patient Register,^4^ Swedish Medical Birth Register^5^ | National Patient Registry,^6^ Medical Birth Registry of Norway^7^ |

## ^a^ https://www.abis-studien.se ^b^ https://www.fhi.no/moba-en

## **Supplementary Table 3.** Data sources of variables used in ABIS and MoBa^a^

|  | ABIS | MoBa |
| --- | --- | --- |
| Outcome |  | |
| Inflammatory bowel disease | >2 hospital-based or inpatient records of K50, K51, K52.3 based on International Statistical Classification of Diseases and Related Health Problems, Tenth Revision (ICD-10) codes for inflammatory bowel disease from national patient registers.^4, 6^ IBD definition also included events with discordant CD and UC diagnoses for the past 5 years of follow-up. IBD unclassified was not used as a separate outcome. | |
| Crohn’s disease | >2 hospital-based or inpatient records of K50 from national patient registers^4, 6^ | |
| Ulcerative colitis | >2 hospital-based or inpatient records of K51 from national patient registers^4, 6^ | |
| Follow-up time | From the start of exposure (1 and 3 years of age) to the first recorded diagnosis or the end of study follow-up (31 December 2020 [ABIS], 31 December 2021 [MoBa]). | |
| Exposure |  |  |
| Diet diversity by 1 year | 12 months questionnaire | 18 months questionnaire |
| Diet diversity by 3 years | 30-36 months questionnaire | 36 months questionnaire |
| Covariates in the adjusted model |  |  |
| Child’s sex (female/male) | Birth questionnaire | Medical Birth Registry of Norway |
| Parental origin (Sweden, Norway/other country) | Birth questionnaire | 15 GW questionnaire |
| Maternal education level (<11, 12, >13 years) | Birth questionnaire | 15 GW questionnaire |
| Parental IBD (yes/no) | Birth questionnaire | Norwegian Patient Registry |
| Maternal comorbidities (type 1 diabetes, rheumatoid arthritis or thyroid disease) | Birth questionnaire | 15 GW questionnaire |
| Covariates in sensitivity analyses |  |  |
| Full breastfeeding duration (<4, 4-6, >6 months) | 12-month questionnaire | 6-month questionnaire |
| Maternal smoking in pregnancy (yes/no) | Birth questionnaire | 15 GW, 30 GW, 6-month questionnaires |
| Delivery mode (vaginal, cesarean) | Birth questionnaire | Medical Birth Registry of Norway |
| Maternal age at delivery | Swedish Medical Birth Register | Medical Birth Registry of Norway |
| Birth weight | Birth questionnaire | Medical Birth Registry of Norway |
| Gestational age | Birth questionnaire | Medical Birth Registry of Norway |
| Child’s diet quality by 1 year^8^ | 12-month questionnaire | 18-month questionnaire |
| Child’s antibiotic use by 1 year | 12-month questionnaire | 18-month questionnaire |
| Child’s intake of sugar-sweetened beverages by 1 year^8^ | 12-month questionnaire | 18-month questionnaire |

^a^ Variables were coded as presented in Table 1. ABIS, All Babies in Southeast Sweden; GW, gestational week; IBD, inflammatory bowel disease; MoBa, The Norwegian Mother, Father and Child Cohort Study.

## **Supplementary Table 4.** Description of the food categorization in the diet diversity score

| ABIS  12 months | MoBa  18 months | ABIS  30-36 months | MoBa  36 months | Food items | Food groups (0-2 points/group) | Diet diversity score (0-10 points) |
| --- | --- | --- | --- | --- | --- | --- |
| Bread | Sandwich with filling (liver paste, meat, fish, cheese, jam/honey, other filling) | Bread | Bread/crispbread | Bread | Grains, roots, tubers | Diet diversity (low, medium, high) |
| Home-made porridge | Home-made  porridge | - | - | Home-made porridge |  |  |
| Prepared porridge, gruel | Instant porridge | - | - | Baby cereals |  |  |
| Potatoes, roots | Potatoes | Potatoes, roots | Potatoes | Potatoes |  |  |
| - | - | Pasta | Pasta, spaghetti, noodles | Pasta |  |  |
| Vegetables, mushrooms | Raw vegetables, cooked vegetables, peas and legumes | Vegetables, mushrooms | Raw vegetables, salad, cooked vegetables | Vegetables | Vegetables |  |
| Fruits, berries | Fruit | Fruits, berries | Fruits | Fruits | Fruits |  |
| Milk | Whole, low-fat, extra low-fat, skimmed milk | Milk | Milk (whole, sweet/sour, low-fat, extra low-fat, skimmed) | Milk | Dairy |  |
| Yoghurt, sour milk | Yoghurt (Lactobacillus, natural, with fruit), sour milk | Yoghurt, sour milk | Yoghurt (natural, with fruit, Lactobacillus) | Yoghurt |  |  |
| Cheese | Cheese sandwich | Cheese | Other types of cheese (not brown cheese) | Cheese |  |  |
| Game, beef, pork/sausage | Meat or liver paste sandwich, meat/sausage/meatballs, etc. | Game, beef, pork/sausage | Meat filling (liver paste, ham), meat/rissoles, sausages etc. | Meat | Animal proteins |  |
| Freshwater fish, fish from Baltic Sea, and other fish | Fish/fish balls/fish pudding, fish sandwich (sardine, mackerel) | Freshwater fish, fish from Baltic Sea, and other fish | Fish filling (mackerel, caviar), oily fish (salmon, herring), white fish (cod, coley), fish pudding/fishcake/fish balls, etc. | Fish |  |  |
| - | - | Eggs | Eggs | Eggs |  |  |

## **Supplementary Table 5.** Follow-up and incidence of IBD, CD and UC in the ABIS and MoBa cohorts,

|  | n event | PYR of follow-up | Incidence rate per 100,000 PYR (95%CI)^a^ | Cumulative incidence (%) at the end of follow-up^a^ | Cumulative incidence (%) by <15 years of age^b^ |
| --- | --- | --- | --- | --- | --- |
| ABIS |  |  |  |  |  |
| IBD | 76 | 234,071 | 32.47 (25.58, 40.64) | 0.69 (0.54, 0.86) | 0.15 (0.08, 0.24) |
| CD | 29 | 234,309 | 12.38 (8.29, 17.78) | 0.26 (0.18, 0.38) | 0.07 (0.03, 0.14) |
| UC | 35 | 234,302 | 14.94 (10.40, 20.78) | 0.32 (0.22, 0.44) | 0.05 (0.02, 0.12) |
| MoBa |  |  |  |  |  |
| IBD | 231 | 1,070,254 | 21.58 (18.89, 24.55) | 0.33 (0.29, 0.37) | 0.24 (0.20, 0.28) |
| CD | 102 | 1,070,822 | 9.53 (7.77, 11.56) | 0.15 (0.12, 0.18) | 0.11 (0.09, 0.14) |
| UC | 62 | 1,070,981 | 5.79 (4.44, 7.42) | 0.09 (0.07, 0.11) | 0.05 (0.04, 0.07) |

^a^End of follow-up was December 31, 2020 (ABIS) and December 31, 2021 (MoBa).

^b^Due to the diffferent follow-up time in ABIS and MoBa, we restricted this to IBD events with onset before 15 years of age.

ABIS, All Babies in Southeast Sweden study; CD, Crohn’s disease; CI, confidence interval; IBD, inflammatory bowel disease; MoBa, The Norwegian Mother, Father and Child Cohort Study; PYR, person-year; UC, ulcerative colitis.

## **Supplementary Table 6.** Study characteristics of participants in ABIS and MoBa presented by non-events and IBD cases by 1 year of age

|  | ABIS | | MoBa | | |
| --- | --- | --- | --- | --- | --- |
|  | All  (n=11,015) | IBD events (n=76) | All (n=70,257) |  | IBD events (n=231) |
| Diet diversity score by 1 year | |  |  |  |  |
| Mean (SD) | 6.3 (1.4) | 6.4 (1.3) | 6.3 (1.3) |  | 6.1 (1.4) |
| Diet diversity score by 3 years | |  |  |  |  |
| Mean (SD) | 6.2 (1.4) | 6.2 (1.2) | 6.4 (1.3) |  | 6.2 (1.4) |
| Child’s sex |  |  |  |  |  |
| Female | 5,290 (48.0) | 35 (46.1) | 35,923 (51.1) |  | 99 (43.0) |
| Male | 5,725 (52.0) | 41 (53.9) | 34,333 (48.9) |  | 131 (57.0) |
| Parental origin^a^ |  |  |  |  |  |
| Sweden/Norway | 9,743 (88.5) | 64 (84.2) | 61,670 (87.8) |  | 207 (90.0) |
| *Missing* | 240 (2.2) | 0 (0.0) | 1,782 (2.5) |  | 3 (1.3) |
| Maternal education level^b^ | |  |  |  |  |
| <11 years | 789 (7.2) | 10 (13.2) | 4,231 (6.0) |  | 15 (6.5) |
| 12 years | 5,972 (54.2) | 44 (57.9) | 19,190 (27.3) |  | 67 (29.1) |
| >13 years | 4,007 (36.4) | 22 (28.9) | 45,977 (65.4) |  | 145 (63.0) |
| *Missing* | 247 (2.2) | 0 (0.0) | 858 (1.2) |  | 3 (1.3) |
| Parental IBD^c^ |  |  |  |  |  |
| Yes | 141 (1.3) | 4 (5.3) | 1,709 (2.4) |  | 7 (3.0) |
| Maternal comorbidities^d^ | |  |  |  |  |
| Yes | 385 (3.5) | 3 (3.9) | 2,853 (4.1) |  | 11 (4.8) |
| Full breastfeeding duration | |  |  |  |  |
| <4 months | 2,676 (24.3) | 23 (30.3) | 27,636 (39.3) |  | 85 (37.0) |
| 4-6 months | 3,402 (30.9) | 23 (30.3) | 30,557 (43.5) |  | 102 (44.3) |
| >6 months | 1,518 (13.8) | 14 (18.4) | 9,444 (13.4) |  | 38 (16.5) |
| *Missing* | 3,419 (31.0) | 16 (21.1) | 2,619 (3.7) |  | 5 (2.2) |
| Maternal smoking in pregnancy | |  |  |  |  |
| Yes | 1,009 (9.2) | 8 (10.5) | 5,516 (7.9) |  | 26 (11.3) |
| *Missing* | 247 (2.2) | 0 (0.0) | 866 (1.2) |  | 2 (0.9) |
| Mode of delivery |  |  |  |  |  |
| Vaginal | 8,907 (80.9) | 59 (77.6) | 59,982 (85.4) |  | 192 (83.5) |
| *Missing* | 868 (7.9) | 6 (7.9) | 0 (0.0) |  | 0 (0.0) |
| Maternal age at delivery^e^ | |  |  |  |  |
| <25 | 1,555 (14.1) | 9 (11.8) | 6,637 (9.4) |  | 20 (8.7) |
| 25-34 | 7,908 (71.8) | 58 (76.3) | 51,056 (72.7) |  | 174 (75.7) |
| 35-44 | 1,359 (12.3) | 8 (10.5) | 12,528 (17.8) |  | 36 (15.7) |
| *Missing* | 193 (1.8) | 1 (1.3) | 35 (0.0) |  | 0 (0.0) |
| Birth weight (g)^f^ |  |  |  |  |  |
| Mean (SD) | 3,580 (543) | 3,589 (553) | 3,571 (578) |  | 3,626 (603) |
| *Missing* | 110 (1.0) | 0 (0.0) | 39 (0.0) |  | 0 (0.0) |
| Gestational age (weeks)^g^ | |  |  |  |  |
| Mean (SD) | 39.7 (1.7) | 39.9 (1.5) | 39.4 (1.9) |  | 39.5 (1.7) |
| *Missing* | 194 (1.8) | 1 (1.3) | 289 (0.4) |  | 1 (0.4) |
| Child’s diet quality by 12 months^h^ | |  |  |  |  |
| Low | 4,551 (41.3) | 37 (48.7) | 21,522 (30.6) |  | 88 (38.3) |
| Medium | 3,437 (31.2) | 25 (32.9) | 26,764 (38.1) |  | 75 (32.6) |
| High | 3,027 (27.5) | 14 (18.4) | 21,970 (31.3) |  | 67 (29.1) |

^a^ Parent’s native language (MoBa), parent’s country of birth (ABIS).

^b^ Education at the time of birth.

^c^ Defined as having at least one parent with IBD.
^d^ Type 1 diabetes (insulin-treated diabetes before or during pregnancy [MoBa] or type 1 diabetes/insulin-treated diabetes [ABIS]), autoimmune thyroid disease, or rheumatoid arthritis.

^e^ <15 years were defined as missing in ABIS (not applicable in MoBa), and >44 years were changed to missing in both cohorts.

^f^ <270 or >6,999 grams were changed to missing. ^j^<22 or >45 weeks were changed to missing.

^g^ <22 or >45 weeks were changed to missing.

^h^ Calculated by using a modified Healthy Eating Index score described by Guo et al. 2024.^8^

ABIS, All Babies in Southeast Sweden; CD, Crohn’s disease; IBD, inflammatory bowel disease; IQR, interquartile range; MoBa, The Norwegian Mother, Father, and Child Cohort Study; SD, standard deviation; UC, ulcerative colitis.

## **Supplementary Table 7.** Tracking of diet diversity from 1 to 3 years of age in children with data at both ages^a^

|  | Children | |  | Change from 1 to 3 years | | |
| --- | --- | --- | --- | --- | --- | --- |
|  | 1 year, n (%) | 3 years, n (%) |  | Decreased diversity, % | Stable  diversity, % | Increased diversity, % |
| Diet diversity |  |  |  |  |  |  |
| ABIS | 11,015 | 8,816 |  | 29.3 | 43.3 | 27.4 |
| Low | 3,653 (33.2) | 2,923 (33.2) |  | n.c | 15.3 | 16.3 |
| Medium | 3,691 (33.5) | 2,984 (33.8) |  | 10.6 | 12.4 | 11.1 |
| High | 3,671 (33.3) | 2,909 (33.0) |  | 18.7 | 15.5 | n.c |
| MoBa | 70,256 | 57,175 |  | 24.6 | 49.4 | 26.0 |
| Low | 23,138 (32.9) | 19,141 (33.5) |  | n.c | 17.9 | 14.3 |
| Medium | 24,056 (34.2) | 17,443 (30.5) |  | 10.3 | 12.4 | 11.7 |
| High | 23,062 (32.8) | 20,591 (36.0) |  | 14.3 | 19.1 | n.c |

^a^ Change in the distribution of food intake from 1 to 3 years was only calculated in those children with data at both time points (n=57,8723). Stability is shown as the percentage of the participants remaining at the same level of diet diversity at each time point. Decrease and increase are presented as percentages of individuals with a decreased or increased diversity in intake over time.

ABIS, All Babies in Southeast Sweden; MoBa, The Norwegian Mother, Father and Child Cohort Study; n.c, no change (decrease/increase) possible.

##

## **Supplementary Table 8.** Cohort-specific hazard ratios for inflammatory bowel disease according to diet diversity at 1 and 3 years of age

|  | **ABIS** | | | | **MoBa** | | | |
| --- | --- | --- | --- | --- | --- | --- | --- | --- |
|  | N | n event | Crude HR (95% CI) | Adjusted HR (95% CI)^a^ | n | n event | Crude HR (95% CI) | Adjusted HR (95% CI) ^a^ |
| Diet diversity at 1 year |  |  |  |  |  |  |  |  |
| Low | 3,631 | 20 | Reference | Reference | 23,139 | 91 | Reference | Reference |
| Medium | 3,682 | 33 | 1.63 (0.94, 2.84) | 1.81 (1.04, 3.15) | 24,056 | 79 | 0.87 (0.64, 1.18) | 0.87 (0.63, 1.18) |
| High | 3,702 | 23 | 1.12 (0.62, 2.05) | 1.26 (0.68, 2.33) | 23,062 | 61 | 0.73 (0.53, 1.01) | 0.73 (0.52, 1.02) |
| Per unit increase |  |  | 1.03 (0.88, 1.20) | 1.06 (0.90, 1.25) |  |  | 0.89 (0.81, 0.98) | 0.89 (0.81 0.98) |
| Diet diversity at 3 years |  |  |  |  |  |  |  |  |
| Low | 2,923 | 19 | Reference | Reference | 19,141 | 81 | Reference | Reference |
| Medium | 2,984 | 23 | 1.19 (0.65, 2.18) | 1.28 (0.69, 2.37) | 17,443 | 50 | 0.70 (0.49, 1.00) | 0.69 (0.48, 1.00) |
| High | 2,909 | 23 | 1.22 (0.67, 2.24) | 1.31 (0.70, 2.45) | 20,591 | 70 | 0.86 (0.62, 1.18) | 0.86 (0.62, 1.20) |
| Per unit increase |  |  | 1.05 (0.87, 1.25) | 1.06 (0.88, 1.27) |  |  | 0.91 (0.82, 1.99) | 0.91 (0.82, 1.00) |

## ^a^ Adjusted for child’s sex, parental IBD, origin, maternal education and comorbidities. CI, confidence interval; HR, hazard ratio.

## **Supplementary Table 9.** Cohort-specific hazard ratios for ulcerative colitis according to diet diversity at 1 and 3 years of age

|  | ABIS | | | | MoBa | | | |
| --- | --- | --- | --- | --- | --- | --- | --- | --- |
|  | N | n event | Crude HR (95% CI) | Adjusted HR (95% CI) ^a^ | n | n event | Crude HR (95% CI) | Adjusted HR (95% CI) ^a^ |
| Diet diversity at 1 year |  |  |  |  |  |  |  |  |
| Low | 3,631 | 9 | Reference | Reference | 23,139 | 29 | Reference | Reference |
| Medium | 3,682 | 11 | 1.21 (0.50, 2.92) | 1.34 (0.57, 3.19) | 24,056 | 18 | 0.64 (0.35, 1.15) | 0.57 (0.31, 1.05) |
| High | 3,702 | 15 | 1.63 (0.71. 3.72) | 1.86 (0.80, 4.34) | 23,062 | 15 | 0.59 (0.32, 1.08) | 0.55 (0.29, 1.04) |
| Per one unit increase |  |  | 1.17 (0.90, 1.52) | 1.23 (0.94, 1.60) |  |  | 0.81 (0.68, 0.96) | 0.78 (0.66, 0.94) |
| Diet diversity at 3 years |  |  |  |  |  |  |  |  |
| Low | 2,923 | 12 | Reference | Reference | 19,141 | 25 | Reference | Reference |
| Medium | 2,984 | 7 | 0.57 (0.22, 1.45) | 0.66 (0.25, 1.71) | 17,443 | 10 | 0.46 (0.22, 0.96) | 0.46 (0.22, 0.97) |
| High | 2,909 | 15 | 1.26 (0.59, 2.70) | 1.45 (0.65, 3.24) | 20,591 | 17 | 0.69 (0.37, 1.29) | 0.65 (0.34, 1.24) |
| Per one unit increase |  |  | 1.05 (0.82, 1.35) | 1.08 (0.84, 1.39) |  |  | 0.80 (0.66, 0.78) | 0.78 (0.65, 0.95) |

## ^a^ Adjusted for child’s sex, parental IBD, origin, maternal education and comorbidities. CI, confidence interval; HR, hazard ratio.

## **Supplementary Table 10.** Cohort-specific hazard ratios for Crohn’s disease according to diet diversity at 1 and 3 years of age

|  | ABIS | | | | MoBa | | | |
| --- | --- | --- | --- | --- | --- | --- | --- | --- |
|  | N | n event | Crude HR (95% CI) | Adjusted HR (95% CI) ^a^ | n | n event | Crude HR (95% CI) | Adjusted HR (95% CI) ^a^ |
| Diet diversity at 1 year |  |  |  |  |  |  |  |  |
| Low | 3,631 | 9 | Reference | Reference | 23,139 | 31 | Reference | Reference |
| Medium | 3,682 | 13 | 1.43 (0.61, 3.34) | 1.66 (0.69, 4.01) | 24,056 | 38 | 1.23 (0.76, 1.98) | 1.23 (0.75, 2.01) |
| High | 3,702 | 7 | 0.76 (0.28, 2.04) | 0.87 (0.31, 2.44) | 23,062 | 33 | 1.15 (0.70, 1.88) | 1.16 (0.70, 1.91) |
| Per one unit increase |  |  | 0.92 (0.73, 1.16) | 0.95 (0.75, 1.21) |  |  | 1.02 (0.88, 1.18) | 1.02 (0.88, 1.19) |
| Diet diversity at 3 years |  |  |  |  |  |  |  |  |
| Low | 2,923 | 4 | Reference | Reference | 19,141 | 36 | Reference | Reference |
| Medium | 2,984 | 9 | 2.20 (0.68, 7.15) | 2.22 (0.70, 7.01) | 17,443 | 22 | 0.69 (0.40, 1.18) | 0.69 (0.40, 1.20) |
| High | 2,909 | 7 | 1.76 (0.52, 6.02) | 1.77 (0.53, 5.88) | 20,591 | 34 | 0.93 (0.58, 1.50) | 0.94 (0.58, 1.53) |
| Per one unit increase |  |  | 1.05 (0.76, 1.45) | 1.05 (0.70, 1.45) |  |  | 0.98 (0.84, 1.15) | 0.99 (0.84, 1.16) |

## ^a^ Adjusted for child’s sex, parental IBD, origin, maternal education and comorbidities. CI, confidence interval; HR, hazard ratio.

## **Supplementary Table 11.** Pooled hazard ratios for inflammatory bowel disease according to diet diversity at 1 and 3 years of age. Sensitivity analyses additionally adjusted for breastfeeding duration, maternal smoking in pregnancy, perinatal factors (including delivery mode, birth weight, gestational age) and child’s exposure to early-life diet quality, antibiotic use, and intake of sugar-sweetened beverages

|  | Adjusted hazard ratio (95% confidence interval)^a^ | | | | | |
| --- | --- | --- | --- | --- | --- | --- |
|  | Breastfeeding | Maternal smoking | Perinatal factors | Diet quality, age 1yr | Antibiotic use | Sugar-sweetened beverages |
| Diet diversity at 1 year |  |  |  |  |  |  |
| Low | Reference | Reference | Reference | Reference | Reference | Reference |
| Medium | 1.12 (0.61, 2.04) | 1.21 (0.60, 2.44) | 1.22 (0.56, 2.64) | 1.30 (0.58, 2.89) | 1.20 (0.58, 2.49) | 1.19 (0.59, 2.41) |
| High | 0.77 (0.57, 1.04) | 0.91 (0.55, 1.49) | 0.87 (0.54, 1.39) | 1.03 (0.52, 2.03) | 0.92 (0.53, 1.61) | 0.89 (0.54, 1.47) |
| Per one unit increase | 0.96 (0.79, 1.15) | 0.96 (0.81, 1.13) | 0.96 (0.80, 1.14) | 0.99 (0.79, 1.24) | 0.96 (0.80, 1.15) | 0.96 (0.81, 1.13) |
| Diet diversity at 3 years |  |  |  |  |  |  |
| Low | Reference | Reference | Reference | Reference | Reference | Reference |
| Medium | 0.83 (0.47, 1.46) | 0.90 (0.51, 1.59) | 0.84 (0.51, 1.39) | 0.93 (0.51, 1.69) | 0.78 (0.53, 1.13) | 0.89 (0.50, 1.60) |
| High | 0.97 (0.58, 1.64) | 0.98 (0.69, 1.39) | 0.93 (0.69, 1.24) | 1.03 (0.72, 1.48) | 0.96 (0.62, 1.48) | 0.97 (0.67, 1.42) |
| Per one unit increase | 0.95 (0.79, 1.15) | 0.96 (0.84, 1.11) | 0.95 (0.84, 1.06) | 0.98 (0.85, 1.13) | 0.95 (0.82, 1.09) | 0.96 (0.83, 1.11) |

## ^a^ Also adjusted for child’s sex, parental IBD, origin, maternal education and comorbidities.

## **Supplementary Table 12.** Cohort-specific hazard ratios for inflammatory bowel disease according to diet diversity at 1 and 3 years of age restricted to a follow-up of <18 years

|  | Adjusted hazard ratio (95% confidence interval) |
| --- | --- |
|  | <18 years |
| Diet diversity at 1 year |  |
| Low | Reference |
| Medium | 1.01 (0.61–1.65) |
| High | 0.91 (0.47–1.76) |
| Diet diversity at 3 years |  |
| Low | Reference |
| Medium | 0.90 (0.47–1.75) |
| High | 1.30 (0.55–3.06) |

**REFERENCES**

1. Nwaru BI, Takkinen HM, Kaila M, et al. Food diversity in infancy and the risk of childhood asthma and allergies. J Allergy Clin Immunol 2014;133:1084-91.

2. Dello Russo M, Formisano A, Lauria F, et al. Dietary Diversity and Its Association with Diet Quality and Health Status of European Children, Adolescents, and Adults: Results from the I.Family Study. Foods 2023;12.

3. Venter C, Maslin K, Holloway JW, et al. Different Measures of Diet Diversity During Infancy and the Association with Childhood Food Allergy in a UK Birth Cohort Study. J Allergy Clin Immunol Pract 2020;8:2017-2026.

4. Ludvigsson JF, Andersson E, Ekbom A, et al. External review and validation of the Swedish national inpatient register. BMC Public Health 2011;11:450.

5. Cnattingius S, Källén K, Sandström A, et al. The Swedish medical birth register during five decades: documentation of the content and quality of the register. Eur J Epidemiol 2023;38:109-120.

6. Bakken IJ, Ariansen AMS, Knudsen GP, et al. The Norwegian Patient Registry and the Norwegian Registry for Primary Health Care: Research potential of two nationwide health-care registries. Scand J Public Health 2020;48:49-55.

7. Irgens LM. The Medical Birth Registry of Norway. Epidemiological research and surveillance throughout 30 years. Acta Obstet Gynecol Scand 2000;79:435-9.

8. Guo A, Ludvigsson J, Brantsæter AL, et al. Early-life diet and risk of inflammatory bowel disease: a pooled study in two Scandinavian birth cohorts. Gut 2024;73:590-600.
